# Fibrin-associated large B-cell lymphoma: first case report within a cerebral artery aneurysm and literature review

**DOI:** 10.1186/s12885-019-6123-1

**Published:** 2019-09-13

**Authors:** Magda Zanelli, Maurizio Zizzo, Marco Montanaro, Vito Gomes, Giovanni Martino, Loredana De Marco, Giulio Fraternali Orcioni, Maria Paola Martelli, Stefano Ascani

**Affiliations:** 1 Pathology Unit, Azienda Unità Sanitaria Locale-IRCCS Reggio Emilia, Reggio Emilia, Italy; 2 Surgical Oncology Unit, Azienda Unità Sanitaria Locale-IRCCS di Reggio Emilia, Reggio Emilia, Italy; 3 Clinical and Experimental Medicine PhD Program, University of Modena and Reggio Emilia, Modena, Italy; 4 Hematology Unit, Ospedale di Belcolle, Viterbo, Italy; 5 Pathology Unit, Ospedale di Belcolle, Viterbo, Italy; 6 Hematology Unit, CREO, Azienda Ospedaliera di Perugia, University of Perugia, Perugia, Italy; 7 Pathology Unit, Azienda Ospedaliera S. Croce e Carle, Cuneo, Italy; 8 Pathology Unit, Ospedale di Terni, University of Perugia, Perugia, Italy

**Keywords:** Fibrin, B-cell, Lymphoma, Epstein-Barr virus

## Abstract

**Background:**

Fibrin-associated diffuse large B-cell lymphoma (FA-DLBCL) is a rare Epstein-Barr virus (EBV) positive lymphoproliferative disorder included in the current World Health Organization (WHO) classification. It arises within fibrinous material in the context of hematomas, pseudocysts, cardiac myxoma or in relation with prosthetic devices. In these clinical settings the diagnosis requires an high index of suspicion, because it does not form a mass itself, being composed of small foci of neoplastic cells. Despite overlapping features with diffuse large B-cell lymphoma associated with chronic inflammation, it deserves a separate classification, being not mass-forming and often following an indolent course.

**Case presentation:**

A 64-year-old immunocompetent woman required medical care for cerebral hemorrhage. Computed Tomography (CT) angiography identified an aneurysm in the left middle cerebral artery. A FA-DLBCL was incidentally identified within thrombotic material in the context of the arterial aneurysm. After surgical removal, it followed a benign course with no further treatment.

**Conclusions:**

The current case represents the first report of FA-DLBCL identified in a cerebral artery aneurysm, expanding the clinicopathologic spectrum of this rare entity. A complete literature review is additionally made.

## Background

In the current WHO classification, diffuse large B-cell lymphoma associated with chronic inflammation (DLBCL-CI) is defined as an EBV-driven neoplasm, occurring in longstanding chronic inflammation in restricted spaces [1]. The prototype is pyothorax-associated lymphoma (PAL) arising in patients with a long history of pyothorax, following artificial pneumothorax as treatment for tuberculosis [1]. Recently, another EBV-related entity has been included among DLBCL-CI, but renamed fibrin-associated diffuse large B-cell lymphoma (FA-DLBCL) because it develops within fibrinous material [1].

It has been reported in association with pseudocysts, cardiac myxoma, valve prosthesis, fibrin thrombus, synthetic tube graft, hydrocele, metallic implants, and chronic subdural hematoma [1–25]. Differently from PAL, it does not form masses, being composed of rare neoplastic cells and it represents often an incidental finding [1]. Whereas PAL follows an aggressive course, the majority of FA-DLBCL behave favorably and may not require therapies other than surgery. Rare cases with persistent or localized recurrent disease have been described [9]. Only one case with a poor outcome has been reported so far [24]. We present the first report of FA-DLBCL incidentally disclosed in a cerebral artery aneurysm, widening the clinicopathological spectrum of this rare entity.

## Case presentation

**Fig. 1:**
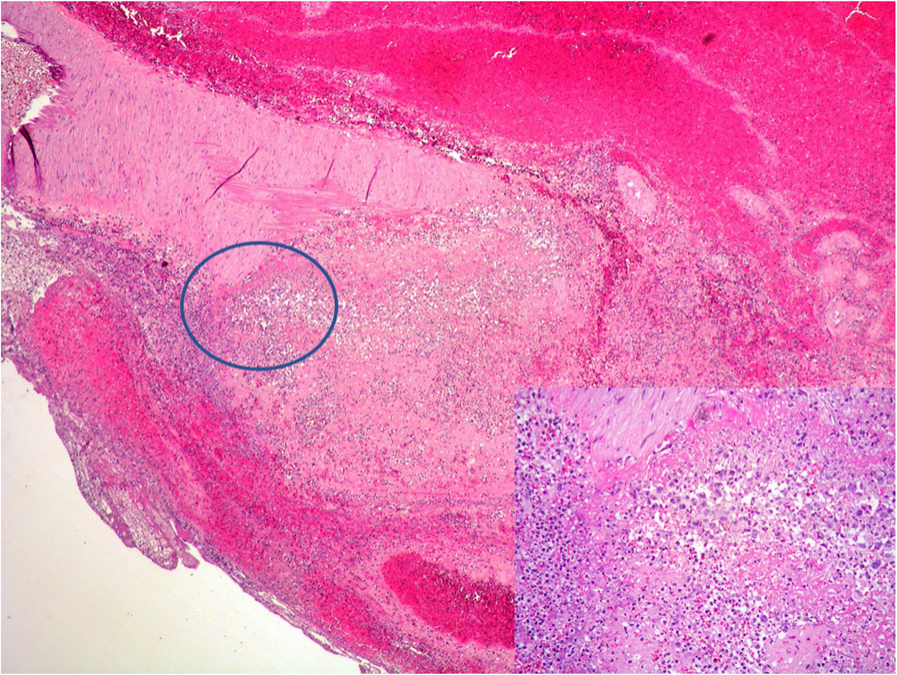
Low power view of artery with interrupted wall and containing thrombotic material (HE 4x); *inset* Rare atypical lymphoid cells lying within the thrombus are recognizable at high power view (HE 20x)

**Fig. 2:**
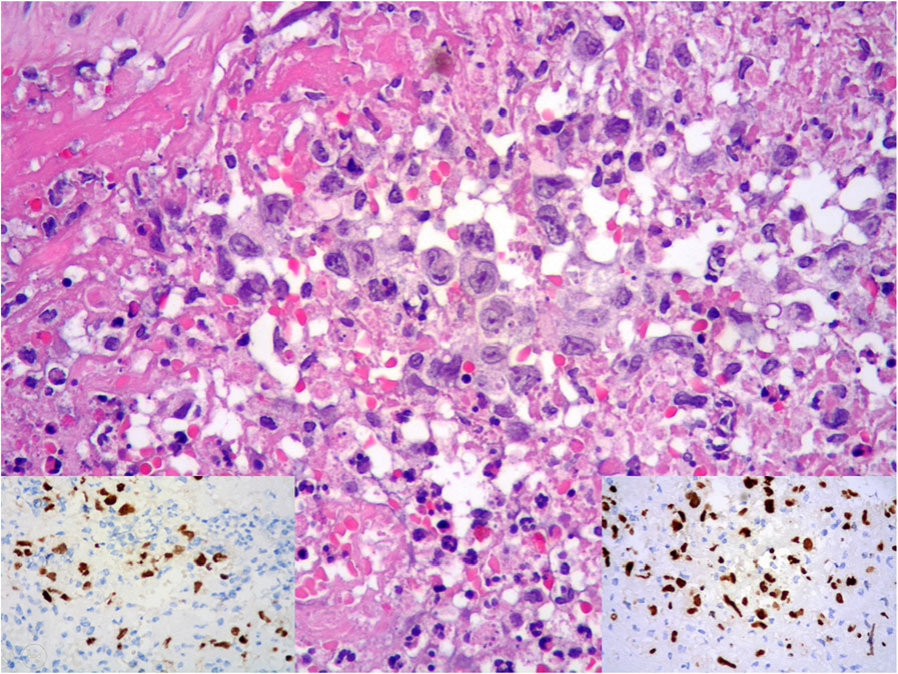
High power detail of large lymphoid cells (HE 40x); *inset left* PAX5 positivity of lymphoid cells; *inset right* MUM1 expression of lymphoid cells

**Fig. 3:**
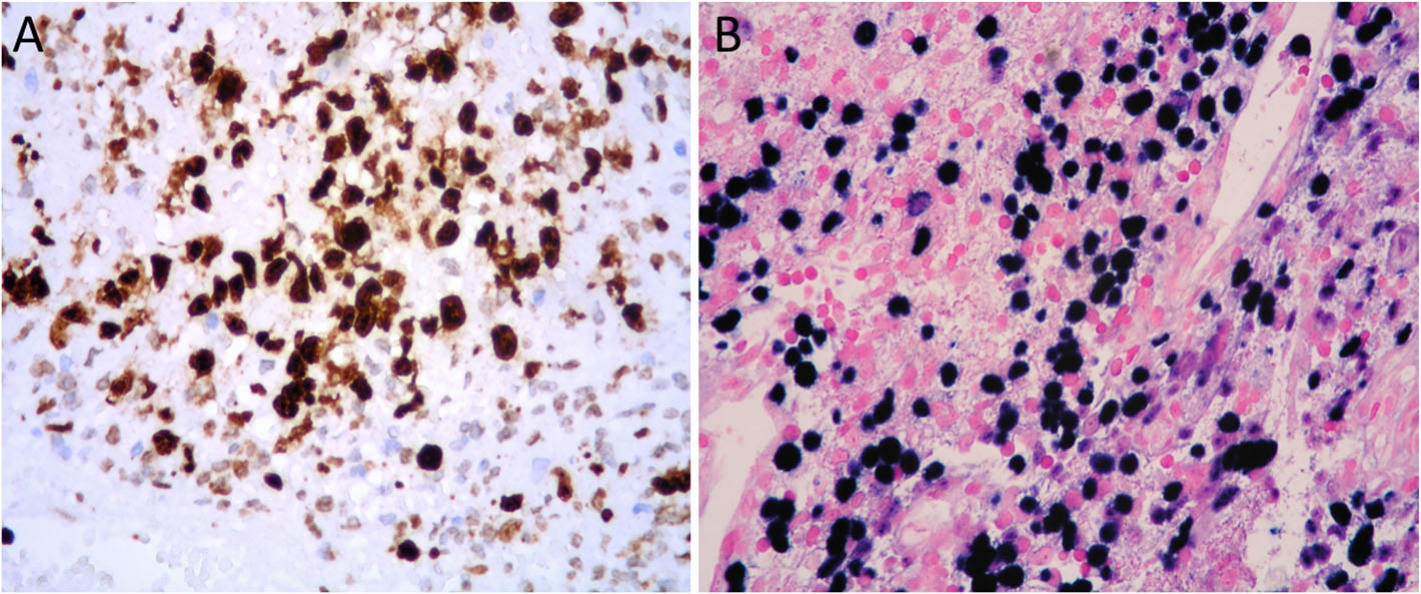
High proliferative index (Ki67) (**a**); Epstein-Barr virus positivity in large-sized cells by in situ hybridization for EBV-encoded RNA (EBER) (**b**)

A 64-year-old immunocompetent woman was referred to hospital for cerebral hemorrhage in left temporal-parietal region. CT angiography detected an aneurysm in the distal segment of left middle cerebral artery. Tiny fragments of brain tissue together with partially organized thrombus were surgically removed. Histologically, it was identified an artery, with an interrupted wall, occluded by thrombotic material (Fig. 1). Small foci of large atypical lymphoid cells (Fig. 1, *inset*; Fig. 2) were disclosed within thrombus. The cells were positive for PAX5 (Fig. 2, *inset left*), CD30 and MUM1 (Fig. 2, *inset right*) with partial expression of CD79α and CD20. The proliferative index (Fig. 3 a) was high (Ki67 about 90%). The cells expressed LMP-1 and were diffusely positive for EBV by in situ hybridization for EBV-encoded RNA (EBER) (Fig. 3, b). Clonal immunoglobulin heavy chain (IGH) rearrangement was detected. A fibrin-associated diffuse large B-cell lymphoma was diagnosed. Staging procedures (CT scan and bone marrow biopsy) were negative. Three months later, CT scan showed an almost complete hemorrhage resorption. No further treatment was given. The patient is alive, free of disease at 8 months from diagnosis.

## Discussion and conclusions

FA-DLBCL is a rare EBV-associated B-cell lymphoma included in the current WHO classification, in the chapter of DLBCL-CI [1]. Differently from DLBCL-CI, it is not mass-forming and therefore disclosed incidentally on histological evaluation of surgical specimens removed for other diseases [1]. Forty seven cases, including our, have been reported so far [1–25]. Clinicopathological data are summarized in Table 1. It shows male predominance with a wide age range. No ethnic differences have been apparently identified so far [9]. All cases, except 2 [9], occurred in immunocompetent individuals, presenting with different symptoms, depending on the underlying conditions in which FA-DLBCL occurred.

**Table 1:** Demographic data, clinical data, and characteristics of reported cases of Fibrin-Associated Diffuse Large B-Cell Lymphoma

SITE/REF.	AGE SEX	Immunosupp	CLINICAL FEATURES	HISTOLOGY	IIC/EBV/CLONALITY	STAGING THERAPY	FOLLOW-UP
*Atrial myxoma* Bagwan 2009 (ref [2])	81/M	negative	Multiple cerebral strokes	Foci of large lymphoid cells at myxoma surface	CD20+, CD79α+, CD10+, BCL6+, BCL2+, CD3-. Ki67:80% EBV: NV. Ig clonality NP.	NS Staging: neg; BM: neg. Surgery+ R-CHOP	NA
*Atrial myxoma* Dimitrova 2010 (ref [3])	51/M	negative	Acute obstructive left heart failure	Foci of large lymphoid cells at myxoma surface	CD20+, CD10+. Ki67 high EBV: NV. Ig clonality NP.	Imaging/BM Staging: neg. Surgery+ CHOP (VI)	NA
*Atrial myxoma* Loong 2010 (ref [4])	70/F	negative	Ischemic stroke	Foci of large lymphoid cells	CD20+, CD79α+, PAX5+, CD43+, MUM1+, CD10-, BCL6+, BCL2+, CD30+, CD138-, HHV8-, CD3-. Ki67 100%. LMP1+, EBNA2+, EBER+. Ig clonality +.	CT/BM Staging: neg. Surgery + R-CEOP (IV)	Died for CH complications (neutropenia+ pneumonia) at 5 mo. No autopsy
*Atrial myxoma* Svec 2012 (ref [5])	60/F	negative	Embolic brain stroke	Foci of large lymphoid cells	CD20+, CD79α+, PAX5+, CD10-MUM1+, CD23+, BCL2+, BCL6-, CD5-, CD3-, cyclin D1-, CD138-, CD38-; Ki67: 100%. LMP1+, EBER+, EBNA2+. FISH MYC, BCL2, BCL6 -. Ig clonality NP.	CT/PET/BM Staging: neg. Surgery+ R-CHOP (VI)	NED at 7 mo
*Atrial myxoma* Bartoloni 2013 (ref [6])	55/F	negative	Fatigue, fever	Foci of large lymphoid cells at myxoma surface	LCA+, CD20+, CD79α+, MUM1+, HHV8-, CD3-, CD5-. Ki67: 90%. LMP1+, EBNA2-, EBER+ Ig clonality NP.	CT/BM staging: neg. Surgery only	NED at 72 mo
*Atrial myxoma* Aguilar 2015 (ref [7])	52/M	negative	Dysarthria and hemiplegia	Foci of large lymphoid cells	CD20+, CD79α+, PAX5+, CD30+, MUM1+, ALK-1-, CD10-CD43-, cyclinD1-, CD3-, LMP1+, EBNA2+, EBER+. Ig clonality +.	CT/BM staging: neg. Surgery only	NED at 42 mo
*Atrial myxoma* Tapan 2015 (ref [8])	49/M	negative	Palpitations	Foci of large lymphoid cells	CD20+, CD79α+, CD30+, MUM1+, CD3-, CD5-, CD10-, CD138-, cyclin D1-, ALK1-, EMA-. Ki67 80%. EBNA2+, EBER+. Ig clonality NP.	NS Staging: neg; BM neg. Surgery + R-CHOP	NED at 12 mo
*Atrial myxoma* Boyer 2017 (ref [9])	54/F	negative	Syncope	Foci of large lymphoid cells	CD20+, PAX5+, CD79α+, BCL6+, CD30+, CD10-, CD138- CD3-, HHV8-, Ki67 80%. EBER+. Ig clonality NP.	NS Staging: neg. Surgery/ Other therapy: NA	NED at 130 mo
*Atrial myxoma* Boyer 2017 (ref [9])	55/F	negative	Syncope, cough, dyspnea	Foci of large lymphoid cells	CD20+, PAX5+, CD79α+, BCL6+, MUM1+, CD10-, CD45+, CD30+, HHV-8-, CD138-, CD3-. Ki67: > 95%. EBER+, LMP1+, EBNA2+. FISH for MYC-. Ig clonality NP.	NS Staging: neg. Surgery only	Died at 2 mo for cardiac cause. Autopsy: No lymphoma
*Atrial myxoma* Boyer 2017 (ref [9])	54/M	negative	Dyspnea, respiratory failure	Foci of large lymphoid cells	CD20+, PAX5+, BCL6+, MUM1+, CD10-, CD38+, CD45+, CD30+, CD3-. Ki67:90% EBER+, LMP1+, EBNA2+. FISH MYC, BCL6, BCL2 -. Ig clonality NP.	CT/BM Staging: neg. Surgery only	Recurrent FA-DLBCL at mitral valve after 25 mo. Died at 26 mo (embolic stroke). No autopsy.
*Atrial myxoma* Yan 2017 (ref [9])	54/M	negative	Congestive heart failure	Foci of large lymphoid cells within fibrin	CD20+, CD79α+, MUM1+, CD10-, BCL6+, CD30+. ALK-, BCL2+, CD3-, CD5-, Ki67 90% LMP1+, EBNA-2+, EBER+, FISH for MYC, Bcl6, BCL2-. Ig clonality NP.	CT/BM Staging: neg. Surgery only	NED at 7 MO
*Atrial myxoma* Yan 2017 (ref [10])	61/F	negative	Congestive heart failure	Foci of large lymphoid cells within fibrin	CD20+, CD79α+, MUM1+, CD10+, BCL6+, CD30+. ALK-, BCL2+, CD3-, CD5-, Ki67 95% LMP1+, EBNA-2+, EBER+, FISH for MYC, Bcl6, BCL2-. Ig clonality NP.	CT/BM Staging: neg. Surgery only	NED at 84 mo
*Atrial myxoma* Yan 2017 (ref [10])	46/F	negative	Congestive heart failure	Foci of large lymphoid cells within fibrin	CD20+, CD79α+, MUM1+, CD10-, BCL6+, CD30+. ALK-, BCL2+, CD3-, CD5-, Ki67 90% LMP1+, EBNA-2+, EBER+, FISH for MYC, Bcl6, BCL2-. Ig clonality NP.	CT/BM Staging: neg. Surgery only	NED at 3 mo
*Atrial myxoma* Yan 2017 (ref [10])	46/F	negative	Congestive heart failure	Foci of large lymphoid cells within fibrin	CD20+, CD79α+, MUM1+, CD10-, BCL6-, CD30+. ALK-, BCL2-, CD3-, CD5-, Ki67 85% LMP1+, EBNA-2+, EBER+, FISH for MYC, Bcl6, BCL2-. Ig clonality NP.	CT/BM Staging: neg. Surgery only	NED at 120 mo
*Atrial thrombus* Qigley 2003 (ref [11])	29/M	negative	Cerebral embolic stroke	Foci of large lymphoid cells at clot’s surface	CD45+, CD20+, CD79α+, CD43+, CD30+, CD3, LMP -, HHV8-, EBER+. Clonality:κ rearrangement +. IGH -, TCR-.	Imaging/BM Staging: neg. Surgery+ R-CHOP (VI)	NED at 24 mo
*Atrial thrombus* Gruver 2012 (ref [12])	56/M	negative	Short breath	Foci of large lymphoid cells within fibrin thrombus	CD20+, CD79α+, PAX5+, CD30+, CD43-, CD45+, BCL6+, MUM1+, BCL2+, CD10-, CD3-, CD5-, HHV8-, MYC + 30%; KI67 > 90% LMP1+, EBNA2 + .EBER+. Ig clonality +.	NS Staging: neg. Surgery+ R-CHOP (VI)	NED at 8 mo
*Myxomatous mitral valve* Gruver 2012 (ref [12])	75/M	negative	Dyspnea, aortic insufficiency, mitral valve regurgitation	Foci of large lymphoid cells within fibrin on mitral valve	CD20+, CD79α+, PAX5+, CD30-, CD43-, CD45+, BCL6-, MUM1+, BCL2+, CD10-, CD3-, CD5-, HHV8-, MYC -. KI67100%. LMP1-, EBNA2-. EBER-. Ig clonality +.	NS Staging: neg. Surgery+R-CVP (I) + R-CHOP (VI)	NED at 39 mo
*Prosthesis (knee)* Cheuk 2005 (ref [13])	78/M	negative	Pain at *knee prosthesis* (implanted 22 yrs. before)	Foci of large lymphoid cells within fibrin and necrosis	CD20+, CD79α+, CD138+/−. CD2-, CD3-, CD5-, CD10-, BCL6-, HHV8-. Ki67:70%. LMP1+, EBER+. Ig clonality +.	NS Staging: neg. Surgery+RT	NED at 24 mo
*Prosthesis (aortic valve)* Bagwan 2009 (ref [2])	50/M	negative	Symptoms of aortic regurgitation. *Aortic valve prosthesis* (16 yrs. before)	Foci of large lymphoid cells within aortic valve leaflets	CD45+, CD20+, CD79α+, CD10+, BC6+/−, BCL2+/−, Ki67:80% LMP1-. Ig clonality: NP.	NS Staging: neg; BM: neg. Surgery+ R-CHOP	Died after 6mo for prosthesis rupture. Autopsy: no lymphoma
*Prosthesis* *(aortic valve)* Berrio 2010 (ref [14])	60/M	negative	Acute left heart failure. History of *aortic valve prosthesis* for stenosis	Foci of large lymphoid cells within valve vegetations	CD20+, CD43+, CD3-. Ki67:80–90% EBV: NV. Ig clonality: NP.	NS Staging: neg. Surgery only	Died for tricuspidal endocarditis, pneumonia 2 yrs. later. No autopsy.
*Prosthesis* *(aortic graft)* Miller 2010 (ref [15])	48/M	negative	Ischemic attack. Marfan sy. Asc.a. aneurysm graft+ aortic valve prosthesis (24 yrs. before)	Foci of large lymphoid cells within fibrin	CD20+, MUM1+, CD10-, BCL6- BCL2+, CD3-, HHV8-. EBER+. Ig clonality +.	CT/PET/BM Staging: neg. Surgery only	NED at 6 mo
*Prosthesis* *(aortic valve)* Miller 2010 (ref [15])	80/F	negative	Heart failure. Aortic valve prosthesis (8 yrs. before)	Foci of large lymphoid cells within fibrin	CD20+, MUM1+, CD10-, BCL6-BCL2-, CD3-, HHV8-, EBER+. Ig clonality +.	CT/PET/BM Staging: neg. Surgery only	Died (for breast cancer 18 mo after aortic valve surgery). No autopsy.
*Prosthesis* *(aortic graft)* Miller 2010 (ref [15])	79/F	negative	Short breath, thoracic pulsing sensation. Tube graft for asc. a. dissection (5 yrs. before)	Foci of large lymphoid cells within fibrin	CD20+, MUM1+, CD10-, BCL6+, BCL2+, CD3-, HHV8-, EBER+, Ig clonality +.	CT/PET/BM Staging: neg. Surgery only	Died for surgical complications. No autopsy
*Prosthesis* *(aortic graft)* Gruver 2012 (ref [12])	55/M	negative	Stroke. Aortic graft for aneurysm (4 yrs. before)	Foci of large lymphoid cells within thrombus	CD20+, CD79α+, PAX5+, CD30+, CD43+, CD45+, BCL6+, MUM1+, BCL2-, CD10-, CD3-, CD5-, HHV8-, MYC-; KI67 100%. LMP1+, EBNA2 + .EBER+. Ig clonality +.	NS Staging: neg. Surgery + R-CEOP (VIII)	NED at 16 mo
*Prosthesis* *(vascular graft)* Boyer 2017 (ref [9])	56/M	negative	IR aorta+ CIA aneurysms. TAA aneurysm graft + thrombectomy (1 yr. before). Asc a. dissection graft (9 yrs. before).	Foci of large lymphoid cells within thrombus of IR aorta and CIA aneurysms. In retrospect foci within thrombus of TAA aneurysm	CD20+, PAX5+, BCL6-, MUM1+, CD10-, CD138-, HHV8-, CD30+, KI67: 95%. EBER+, LMP1+, EBNA2+. FISH for MYC -. Ig clonality +.	CT/PET/BM Staging: neg. Surgery+ R-CHOP (VI) + IT MTX	AWSD at 24 mo. Surgical revision of aortic graft: persistent foci of EBV+ large B cell.
*Prosthesis (vascular graft)* Boyer 2017 (ref [9])	68/M	negative	Lower limbs ischemia. AA aneurysm repair with IR graft (7 yrs. before).	Foci of large lymphoid cells within thrombus	CD20+, PAX5+, BCL6+, CD10-MUM1+, CD30+, HHV8-, KI67 90%, EBER+, LMP1+, EBNA2+. FISH for MYC -. Ig clonality NP.	CT/PET Staging neg. 3 mo after: PET/CT/biopsy: foci of EBV+ cells near adrenal gland. R-COEP (II)	Died at 10 mo for embolic stroke. No progressive lymphoma. No autopsy
*Prosthesis (vascular)* Boyer 2017 (ref [9])	71/M	MG for THY treated with surgery+ steroids+ AZA	AF graft (6 yrs. before).	Foci of large lymphoid cells within thrombus associated with graft	CD20+, CD79α+, PAX5+, CD10-BCL6+, MUM1+, CD30+, CD45+, CD138-, HHV8-, KI67 > 95%, EBER+, LMP1+. Ig clonality +.	NS Staging: neg. Surgery only	NED at 10 mo
*Pseudocyst* *(kidney)* Lee 2009 (ref [16])	61/M	negative	Renal cyst (for 20 yrs)	Foci of large lymphoid cells within necrosis	CD22+, CD45+, CD79α+, MUM1+, PAX5+, CD3-, CD10-, CD20-, CD138-, BCL6-, ALK1-, HHV8-, κ-, λ-, EBER+. Ig clonality NP.	Staging NA. Surgery+ CHOP (VI)	NA
*Pseudocyst* *(spleen)* Loong 2010 (ref [4])	29/M	negative	Abdominal pain	Foci of large lymphoid cells within necrosis	CD20+, CD79α+, PAX5+, CD43+, MUM1+, CD10-, BCL6-CD138-, BCL2+, CD30-, HHV8-, CD3-. Ki67 90%. LMP1+, EBNA2+, EBER+. Ig clonality +.	PET/BM Staging: neg. Surgery (splenectomy) + R (IV)	NED at 6 mo
*Pseudocyst* *(kidney)* Valli 2011 (ref [17])	46/M	negative	Left-sided flank pain	Foci of large lymphoid cells within necrosis	CD20+, MUM1+, CD10-, BCL6-BCL2+, CD30-, HHV8-;Ki67:90%. EBER+. Ig clonality NP.	CT/PET/BM Staging: neg. Surgery+ R-CHOP (VI)	NED at 1 mo
*Pseudocyst* *(adrenal gland)* Boroumand 2012 (ref [18])	63/F	negative	Right abdominal pain	Foci of large lymphoid cells within fibrin	CD20+, CD79α+, PAX5+, MUM1+, BCL2+, CD3-, CD10-, CD30-, BCL6-, HHV8-. Ki67 > 90%. LMP1-; EBER+. Ig clonality NP.	NS Staging: neg. Surgery + R-CHOP (VI) + RT	NED at 40 mo
*Pseudocyst* *(testis)* Boroumand 2012 (ref [18])	27/M	negative	R. scrotal swelling. Herniorraphy followed by l. scrotal hematoma (removed 3 yrs. before)	Foci of large lymphoid cells within fibrin	CD20+, CD79α+, CD30+, MUM1+, BCL2+, CD3-, CD10- BCL6-, HHV8-. Ki67 > 90%. LMP1+, EBER+. Ig clonality NP.	NS Staging: neg. Surgery only	NED at 9 mo
*Pseudocyst (spleen)* Boyer 2017 (ref [9])	37/F	negative	Splenic mass (9 cm), incidentally found	Foci of large lymphoid cells within fibrin	CD20+, PAX5+, MUM1+, CD10-BCL6-, CD30-, CD45+, KI67 > 90% EBER+. Ig clonality NP.	CT/PET/BM Staging: neg. Surgery + R-CHOP (III)	NED at 32 mo
*Pseudocyst (retrop.)* Boyer 2017 (ref [9])	73/M	negative	Femoral a. aneurysm repair	Foci of large lymphoid cells within fibrin	CD20+, PAX5+, CD79α+, BCL6-, CD10-, MUM1+, CD30+, CD45+, HHV8-, KI67 > 95%, EBER+. Ig clonality NP.	CT/BM Staging: neg. Surgery+ R-CHOP (VI)	NED at 43 mo
*Pseudocyst (adrenal gland)* Boyer 2017 (ref [9])	70/M	negative	Adrenal mass (7 cm) causing bladder obstruction	Foci of large lymphoid cells within fibrin	CD20-, PAX5+, CD79α+, BCL6-, CD10-, MUM1+, CD45+, CD30+, CD138-, HHV8-, KI67 > 90%, LMP1-, EBNA2+, EBER+. FISH for MYC -. Ig clonality NP.	CT/PET Staging: neg. Surgery only	NED at 14 mo
*Pseudocyst (retrop.)* Boyer 2017 (ref [9])	44/M	negative	Right flank pain	Foci of large lymphoid cells within fibrin	CD20+, PAX5+, CD10-, BCL6-, MUM1+, CD45+, CD30-, KI67 40%, LMP1+, EBNA2+, EBER+, FISH for MYC -. Ig clonality +.	BM/imaging Staging: neg. 5-CHOP	NED at 84 mo
*Pseudocyst* *(adrenal gland)* Zanelli 2019 (ref [19])	71/F	negative	Lower limbs edema+ abdominal distension	Foci of large lymphoid cells within fibrin	CD20+, PAX5+, CD30+, MUM1+, CD10-, BCL6-, EBER+, Ki67 90%. Ig clonality NP.	CT Staging: neg. Surgery only	NED at 6 mo
*Teratoma* *(ovary)* Valli 2014 (ref [20])	56/F	negative	Abdominal pain+ swelling	Foci of large lymphoid cells	CD20+, MUM1+, CD45+, PAX5+, CD30-, BCL6-, CD10-, CD3-, CD2-, HHV8-, CD138-. Ki67: 80%. EBER+. Ig clonality +.	CT/PET Staging: neg. Surgery+ R-CHOP (VI)	NED at 8 mo
*Hydrocele* *(testis)* Loong 2010 (ref [4])	88/M	negative	Fever, scrotal pain, swelling	Foci of large lymphoid cells within necrosis	CD20+, CD79α+, PAX5+, MUM1+, CD10-, BCL6-, CD138-, BCL2+, CD30-, HHV8-, CD3+, CD2-, CD5-, CD7-. Ki67 70% LMP1+, EBNA2+, EBER+. Ig clonality -.	Staging NA. Surgery only (Orchidectomy)	NA
*Hematoma* *(testis)* Boyer 2017 (ref [9])	79/M	negative	Testicular trauma (5 yrs. before)	Foci of large lymphoid cells within hematoma	CD20+, PAX5+, CD79α+, CD10-CD138-, BCL6-, MUM1+, CD45+, CD30+, HHV8-, KI67 > 90%, EBER+, LMP1+, EBNA2+. Ig clonality +.	NS Staging: neg. Surgery only	NED. Died at 17 mo
*Hematoma (thigh)* Hayes 2014 (ref [21])	91/M	negative	Thigh hematoma. (6 yrs. before leg amputation for popl. a. aneurysm rupture at prior artery bypass graft site)	Foci of large lymphoid cells	CD45+, CD20+, MUM1+, CD30+, CD43+, BCL2+/−, MYC+, p53+/−, HHV8-, CD3-, CD5-, CD10-, BCL1-, BCL6-. Ki67: 90%. LMP1-, EBER+. Ig clonality NP.	NS Staging: neg. Surgery only	NED at 18 mo
*Subdural hematoma* Reyes 1990 (ref [22])	56/M	negative	Headaches, dizziness, unsteady gait	Foci of large lymphoid cells within fibrin, clots, necrosis	B-cell phenotype. EBV NV. Ig clonality NP.	CT/BM Staging: neg. Surgery only	NA
*Subdural hematoma* Sugita 2012 (ref [23])	77/M	negative	Dementia due to head trauma (20 yrs. before)	Foci of large lymphoid cells	CD20+, CD79α+, MUM1+, CD3-, BCL6-, CD10-. Ki67 high. EBNA2+, LMP1-, EBER+. Ig clonality - (rare neoplastic foci).	Imaging Staging: neg. Surgery only	NA
*Subdural hematoma* Kameda 2015 (ref [24])	96/M	negative	Gait disturbs+ anorexia. Trauma+ subdural hematoma (7 mo before).	*Brain mass: DLBCL EBV+. Subdural hematoma: FA-DLBCL.* No continuity among 2 lesions	CD20+, CD79α+, CD3-, CD4-, CD7-, CD8-, LMP1+, EBNA2+, EBER+. Ig clonality NP.	CT Staging: neg at presentation. Brain mass + subdural hematoma resection. IT MTX + cytarabine+ glucocorticoids	Died after 3 mo for lymphoma dissemination. No autopsy
*Subdural hematoma* Boyer 2017 (ref [9])	25/M	negative	SD hematoma since child. Hydrocephalus+ SD catheter. Steroid tp for pituitary overactivity	Foci of large lymphoid cells within hematoma	CD20+, PAX5+, MUM1+, CD10-BCL6-, CD30+, HHV8-, KI67 > 90%; EBER+, LMP1+. Ig clonality NP.	CT/PET/BM Staging: neg. Surgery only	NED at 7 mo
*Arachnoid cyst* Kirshenbaum 2017 (ref [25])	81/M	negative	Tremor, gait ataxia, memory disturbs	Foci of large lymphoid cells within fibrin	CD20+ CD30+, BCL2+, MUM1+, BCL6+/−, CD10-, TdT-, CD5-, cMYC+ (50%) Ki67: > 80%. EBER+. FISH MYC -. Ig clonality +.	CT/PET staging: neg. Surgery (cyst excision) + R-lenalidomide	NA
*Cerebral artery aneurysm* Present case	64/F	negative	Cerebral hemorrhage. Left middle cerebral artery aneurysm	Foci of large lymphoid cells within fibrin	CD20+/−, PAX5+, CD79α+, CD30+, MUM1+, CD10-, BCL6-, EBER+, Ki67 90%. Ig clonality NP.	CT/BM Staging: neg. Surgery only	NED at 5 mo

Literature review of fibrin-associated diffuse large B-Cell Lymphoma

*A* artery, *AA* abdominal aorta, *AF*, aortofemoral, *Asc. A* ascending aorta, *AWSD* alive with stable disease, *AZA* azathioprine, *BM* bone marrow, *CEOP* cyclophosphamide, etoposide, oncovin, prednisone, *CHOP* cyclophosphamide, doxorubicin, vincristine, prednisone, *retro* retroperitoneum, *CIA* common iliac arteries, *CH* chemotherapy, *CVP* cyclophosphamide, vincristine, prednisone, *CT* Computerized tomography, *DEXA* dexamethasone, *F* female, *IT* intrathecal, *IR* infrarenal, *Ig* immunoglobulin, *IGH* immunoglobulin heavy chain, *mo* months, *M* male, *MTX* methotrexate, *MG* myasthenia gravis, *NA* not available, *NED* not evidence of disease, *Neg* negative, *NP* not performed, *NS* not specified, *PBL* plasmablastic lymphoma, *popl. A* popliteal artery, *R* rituximab, *Retrop* retroperitoneum, *RT* radiotherapy, *SD* subdural, *sy* syndrome, *TAA* thoracoabdominal aorta, *TCR* T cell receptor, *THY* Thymoma, *Tp* therapy, *yrs*. years

Cardiac myxoma represents one of the most frequent site of occurrence with 14 cases identified, whereas only occasional cases arose in atrial thrombi and within mixomatous valve degeneration. Some cases have been identified in association with prosthetic devices such as endovascular graft, cardiac valve prosthesis and metallic implant. Time from placement of devices to lymphoma diagnosis is extremely variable, ranging from 1 to more than 20 years. A rather frequent site of presentation is represented by pseudocysts, with a total of 10 cases, in different organs (adrenal gland, spleen, kidney, retroperitoneum, testis). Single descriptions at unusual sites as within testicular hydrocele, ovarian teratoma and testicular hematoma are also reported. The intracranial location appears to be rare, with only 4 cases within chronic subdural hematomas [9, 22–24] and 1 within an arachnoid cyst [25]. Our case represents the first report in a patient with a brain hemorrhage and incidentally identified within thrombotic material in a cerebral artery aneurysm. Notably in all cases evaluated (45/47) staging workup at diagnosis revealed no other sites of disease.

Histologically all cases were remarkably similar and found incidentally, being composed of microscopic foci of large lymphoid cells, embedded within fibrin and not invading adjacent tissue structures. Most cases had a non-germinal center B-cell phenotype and high proliferative index. A strong association with EBV infection is present; as 41/43 evaluated were positive for EBV by EBER-ISH. Notably a type III EBV latency profile, with positivity for LMP-1 and Epstein-Barr nuclear antigen-2 (EBNA-2) was found in most cases (18/22 tested). Type III latency of EBV infection is the hallmark of lymphoproliferative disorders arising in the setting of severe immunosuppression. EBV-infected cells expressing EBNA-2 do not survive in immunocompetent individuals, because destroyed by cytotoxic T-lymphocytes. As patients with FA-DLBCL are immunocompetent, it has been assumed that the restricted environment where FA-DLBCL occurs, allows the EBV-infected B-cells to escape T-cell surveillance [9]. Clonal immunoglobulin rearrangement was identified in most cases evaluated. None of the cases tested by fluorescence in situ hybridization (FISH) showed c-MYC, BCL6 and/or BCL2 rearrangements or amplifications: a rather striking difference from PAL, presenting MYC amplification in 80% of cases [9]. Clinical course of FA-DLBCL is commonly indolent. Remarkably of 36 cases with available follow-up, 30 pursued a benign course, with no evidence of disease from 1 to 130 months. Treatment is variable, although surgery alone often represents the treatment of choice. Sixteen/30 cases were treated with surgery alone, 11 with surgery plus chemotherapy, 1 with surgery plus radiotherapy, 1 with surgery plus immunotherapy, and 1 with surgery plus chemotherapy and radiotherapy. All cases arising within pseudocysts behaved favorably. Local recurrences or persistent disease were seen only in isolated cases in which the primary disease had arisen either within an atrial myxoma (1) or at sites of previous vascular graft (2) [9]. The recurrent or persistent disease presented close to the site initially involved. Two/3 patients died of thromboembolic disease and 1 is alive with stable and localized disease. It has been hypothesized that FA-DLBCL arising at cardiac or vascular sites can recur or persist more easily than cases occurring in sites more amenable to complete surgical removal [9]. Kameda et al reported the unique case with an aggressive course, occurring in an elderly patient within a chronic subdural hematoma observed conservatively [1, 24]. Seven months later, a de novo brain mass developed beneath the hematoma [24]. After surgical removal, the neoplasm within the subdural hematoma appeared consistent with FA-DLBCL and the brain mass was an EBV-positive DLBCL [24]. The authors hypothesized that the lymphoid process developed in the hematoma before infiltrating the brain parenchyma [24]. Once the lymphoma infiltrates outside the subdural hematoma, the prognosis becomes poor [1]. FA-DLBCL shares similarities with breast implant-associated anaplastic large B-cell lymphoma (BIA-ALCL), although the latter is a T/null lymphoma, not EBV-related [1]. Both entities portend a worse prognosis, when infiltrate the surrounding tissues outside the restricted space of origin.

Our case arose in a previously unreported setting, being identified in a cerebral artery aneurysm of a patient with a brain hemorrhage. The disease was totally confined within thrombotic material occluding the artery. After surgical removal, it pursued a benign course with no additional treatment.

In conclusion, FA-DLBCL is a rare EBV-related lymphoproliferative disorder, arising within fibrinous material in different clinical settings. Intracranial location is very rare. This represents the first report within a cerebral artery aneurysm. Diagnosis can be tricky, being FA-DLBCL not mass-forming and composed of tiny neoplastic foci. Clinical behavior is mostly indolent. The limited number of FA-DLBCL reported so far makes difficult to draw definitive conclusion regarding the best treatment. Further cases with longer follow-up would help to adopt the most appropriate therapeutic options for each individual patient.

## Data Availability

All the original data supporting our research are described in the Case presentation section and in the figures’ legends.

## Abbreviations

BIA-ALCL: Breast implant-associated anaplastic large B-cell lymphoma
CT: Computed Tomography
DLBCL-CI: Diffuse large B-cell lymphoma associated with chronic inflammation
EBER: EBV-encoded RNA
EBV: Epstein-Barr virus
FA-DLBCL: Fibrin-associated diffuse large B-cell lymphoma
PAL: Pyothorax-associated lymphoma
WHO: World Health Organization
